# Genome Sequence of a SARS-CoV-2 Strain from Bangladesh That Is Nearly Identical to United Kingdom SARS-CoV-2 Variant B.1.1.7

**DOI:** 10.1128/MRA.00100-21

**Published:** 2021-02-25

**Authors:** Mohammad Enayet Hossain, M. Mahfuzur Rahman, M. Shaheen Alam, Yeasir Karim, Ananya Ferdous Hoque, Sezanur Rahman, Mohammed Ziaur Rahman, Mustafizur Rahman

**Affiliations:** a Virology Laboratory, International Centre for Diarrhoeal Disease Research, Bangladesh, Dhaka, Bangladesh; DOE Joint Genome Institute

## Abstract

The coding-complete genome sequence of a coronavirus strain, SARS-CoV-2/human/BGD/G039392/2021, obtained from a symptomatic male patient with coronavirus disease 2019 (COVID-19) in Dhaka, Bangladesh, is reported. The strain G039392 is 99.9% identical to the UK variant B.1.1.7.

## ANNOUNCEMENT

Severe acute respiratory syndrome coronavirus 2 (SARS-CoV-2) is a positive-sense single-stranded RNA virus from the *Betacoronavirus* genus of the broad family *Coronaviridae*. In Bangladesh, SARS-CoV-2, the causative agent of the coronavirus disease 2019 (COVID-19) pandemic, was first reported on 8 March 2020. Here, we report the complete sequence of SARS-CoV-2 strain G039392, which was identified on 6 January 2021 from a 50-year-old symptomatic male patient in Dhaka, Bangladesh, and is 99.9% identical to the UK variant B.1.1.7.

As part of the countrywide COVID-19 laboratory network, the International Centre for Diarrhoeal Disease Research, Bangladesh (icddr,b), in collaboration with the Government of Bangladesh, has been testing for SARS-CoV-2 since March 2020. Due to the recent emergence of novel variants from the United Kingdom, South Africa, and Brazil (1), we started monitoring SARS-CoV-2 variants. Between 15 December 2020 and 21 January 2021, a total of 5,250 nasopharyngeal swab samples were screened for SARS-CoV-2 by real-time reverse transcriptase PCR using RdRp (open reading frame 1ab [ORF1ab]) and N gene-specific primers and probes (2). The iTaq universal probes one-step kit (Bio-Rad Laboratories, CA, USA) was used in the Bio-Rad CFX96 Touch real-time PCR system. Threshold cycle (*C_T_*) values of ≤37 were considered positive. Of 988 positive samples, 191 were selected (*C_T_* values of ≤25) for variant surveillance by amplification of the spike protein gene using Sanger sequencing. Sequence data for the spike protein gene showed that the spike protein of one strain was nearly identical to the spike protein of the UK variant, and the strain was considered for complete genome sequencing. In brief, total RNA was extracted from nasopharyngeal swab samples using the chemagic viral NA/gDNA kit (PerkinElmer, MA, USA), and reverse transcription was performed using the high-capacity cDNA reverse transcription kit (Thermo Fisher Scientific, CA, USA) following the manufacturer’s protocol. Ninety-eight gene segments were amplified by GoTaq G2 Hot Start *Taq* polymerase (Promega Corp., WI, USA) with specific primer sets (3) to cover the whole genome. All PCR products were analyzed by agarose gel electrophoresis with SYBR Safe staining (Thermo Fisher Scientific) and purified using the ExoSAP‐IT kit (Affymetrix, OH, USA). Sequencing was performed in an Applied Biosystems 3500XL genetic analyzer using the BigDye Terminator v3.1 cycle sequencing ready reaction kit (PerkinElmer). The chromatogram sequencing files were inspected using Chromas v2.23 (Technelysium, QLD, Australia), and the consensus sequences were prepared using SeqMan II (DNASTAR, WI, USA). Multiple sequence alignment was performed using the BioEdit v7.2 program (4).

The assembled SARS-CoV-2/human/BGD/G039392/2021 viral genome consists of 29,796 nucleotides (GC content, 37.98%) with 99.9% coverage, compared with reference strain Wuhan-Hu-1 (GenBank accession number NC_045512). The genome was also compared with UK variant VOC-202012/01 (GISAID accession number EPI_ISL_601443), which indicated that Bangladeshi strain G039392 had all 23 mutations that were identified in UK variant B.1.1.7 (Table 1). In addition, G039392 contained two nonsynonymous mutations (P78S and K460R) in the helicase protein and one nucleotide deletion (A28274^ [with ^ denoting the deleted amino acid]) at the start position of the N gene that were absent in the UK variant. However, the deletion (A28274^) might have no impact on translation of the N gene because three adenine residues were present at the same position (AAA28271), which can be used to initiate translation with the start codon (ATG).

**TABLE 1:** Comparison of mutations between the UK and Bangladeshi strains

		Data for^*b*^:	
Gene segment (position) and nucleotide^*a*^	Amino acid^*a*^	UK strain	Bangladeshi strain
5′ untranslated region			
C241T	−	Y	Y
ORF1a (266 to 13483)			
C913T	−	Y	Y
C3037T	−	Y	Y
C3267T	T1001I	Y	Y
T5266A	−	N	Y
C5388A	A1708D	Y	Y
C5986T	−	Y	Y
T6954C	I2230T	Y	Y
TCTGGTTTT11288^	SGF3675^	Y	Y
RdRp (13442 to 13468, 13468 to 16236)			
C14408T	P323L	Y	Y
C14676T	−	Y	Y
C15279T	−	Y	Y
T16176C	−	Y	Y
Helicase (16237 to 18039)			
C16468T	P78S	N	Y
A17615G	K460R	N	Y
S (21,563 to 25,384)			
ACATGT21765^	HV69^	Y	Y
TTA21991^	Y144^	Y	Y
A23063T	N501Y	Y	Y
C23271A	A570D	Y	Y
A23403G	D614G	Y	Y
C23604A	P681H	Y	Y
C23709T	T716I	Y	Y
T24506G	S982A	Y	Y
G24914C	D1118H	Y	Y
ORF8 (27,894 to 28,259)			
C27972T	Q27*	Y	Y
G28048T	R52I	Y	Y
A28111G	Y73C	Y	Y
N (28,274 to 29,533)			
A28274^	−	N	Y
GAT28280CAT	D3L	Y	Y
GGG28881AAC	RG203KR	Y	Y
T28924G	−	N	Y
C28977T	S235F	Y	Y

a The positions and changes are indicated by comparison with the Wuhan-Hu-1 strain. ^, deletion; *, stop codon; −, synonymous mutation.

b Y, present; N, absent.

The availability of genomic data for the circulating SARS-CoV-2 isolates from different parts of the world will serve as a valuable resource for monitoring emerging new variants.

### Data availability.

The sequence has been deposited in the GenBank database under the accession number MW531680. The GISAID EpiCoV coronavirus SARS-CoV-2 database (www.gisaid.org) accession number for the sequence is EPI_ISL_890237.
